# Seasonality of agricultural exposure as an important predictor of seasonal yellow fever spillover in Brazil

**DOI:** 10.1038/s41467-021-23926-y

**Published:** 2021-06-15

**Authors:** Arran Hamlet, Daniel Garkauskas Ramos, Katy A. M. Gaythorpe, Alessandro Pecego Martins Romano, Tini Garske, Neil M. Ferguson

**Affiliations:** 1 MRC Centre for Global Infectious Disease Analysis; and the Abdul Latif Jameel Institute for Disease and Emergency Analytics, School of Public Health, Imperial College London, London, UK; 2 Secretariat for Health Surveillance, Brazilian Ministry of Health, Brasilia, Brazil

**Keywords:** Viral epidemiology, Viral infection, Risk factors, Agriculture

## Abstract

Yellow fever virus (YFV) is a zoonotic arbovirus affecting both humans and non-human primates (NHP’s) in Africa and South America. Previous descriptions of YF’s seasonality have relied purely on climatic explanations, despite the high proportion of cases occurring in people involved in agriculture. We use a series of random forest classification models to predict the monthly occurrence of YF in humans and NHP’s across Brazil, by fitting four classes of covariates related to the seasonality of climate and agriculture (planting and harvesting), crop output and host demography. We find that models captured seasonal YF reporting in humans and NHPs when they considered seasonality of agriculture rather than climate, particularly for monthly aggregated reports. These findings illustrate the seasonality of exposure, through agriculture, as a component of zoonotic spillover. Additionally, by highlighting crop types and anthropogenic seasonality, these results could directly identify areas at highest risk of zoonotic spillover.

## Abstract (web summary)

Yellow fever virus (YFV) is an arbovirus affecting humans and non-human primates (NHPs) with seasonal transmission. Here Hamlet *et al*. model the monthly occurrence of YF in humans and NHPs across Brazil and show that seasonality of agriculture is an important predictor of seasonal YF transmission.

## Introduction

Yellow fever (YF) is a zoonotic arbovirus affecting both humans and non-human primates (NHP’s) in Africa and South America^1^. In South America the virus is described in two cycles, the sylvatic and the urban. In the sylvatic cycle transmission is maintained between NHP’s via sylvatic mosquito species such as those of the *Haemogogus* and *Sabethes* genera^1^, with humans considered incidental hosts that likely do not contribute to onward transmission. If the virus establishes itself in the urban and diurnal *Aedes aegypti*, the vector of dengue and zika, then transmission can be sustained in the absence of an NHP reservoir host and can lead to rapid and devastating epidemics^2,3^.

In Brazil, since 1942, all cases of YF have been recorded as due to the sylvatic cycle, with much of this transmission confined to the North and North West of the country^4^. However, since 1998 there has been a significant expansion of the risk areas^4^, culminating in the largest outbreaks of YF since the sylvatic cycle was described in the 1930s. As a consequence of a re-emergence process started in 2014, when the virus spread outside the Amazon region (endemic zone)^5^, the densely populated South-Eastern states of the country were strongly affected in 2016–2017, including areas with no record of the disease for decades^6^. This was followed by an equally large and widespread outbreak during the following season, 2017–2018, with additional, low level transmission detected outside its endemic zone in the 2018–2019 season.

While the seasonality of YF has been previously highlighted^7,8^, there remain substantial knowledge gaps about the processes behind this. Seasonal variations in climate can lead to increased vector populations and the suitability for disease transmission, factors which have been used to explain this temporal variability—and even allowed for the forecasting of coming seasons with a high degree of accuracy^9–13^. However, due to sylvatic transmission driving YF cases in humans in Brazil, there remains a counterpart to the seasonality of transmission, the seasonality of exposure. In Brazil around 45% of cases of YF occur in those involved in agriculture or extractivism, both highly seasonal activities^14^. Despite the relationship between agriculture and human disease transmission being one of considerable scientific interest^15,16^, with numerous articles on how landscape changes can affect exposure to human populations^17,18^, changes in vector composition^19,20^ or alter zoonotic reservoir host behaviours^21^, research on how disease transmission is altered by the seasonality of agriculture is lacking.

In this study we seek to investigate the drivers of seasonal YF transmission in Brazil in both humans and NHP ’s. We apply random forest models to predict occurrence of human or NHP YF using covariates related to the seasonality of climate and of agriculture. We assumed that seasonality of agriculture (e.g. harvesting) is a proxy for risk of exposure to the sylvatic cycle. We evaluate the relative importance of these components and identify individual crop types and agricultural activities that are related to increase YF reporting.

## Results

### Seasonality of YF reports in humans and NHP’s in Brazil

**Fig. 1:**
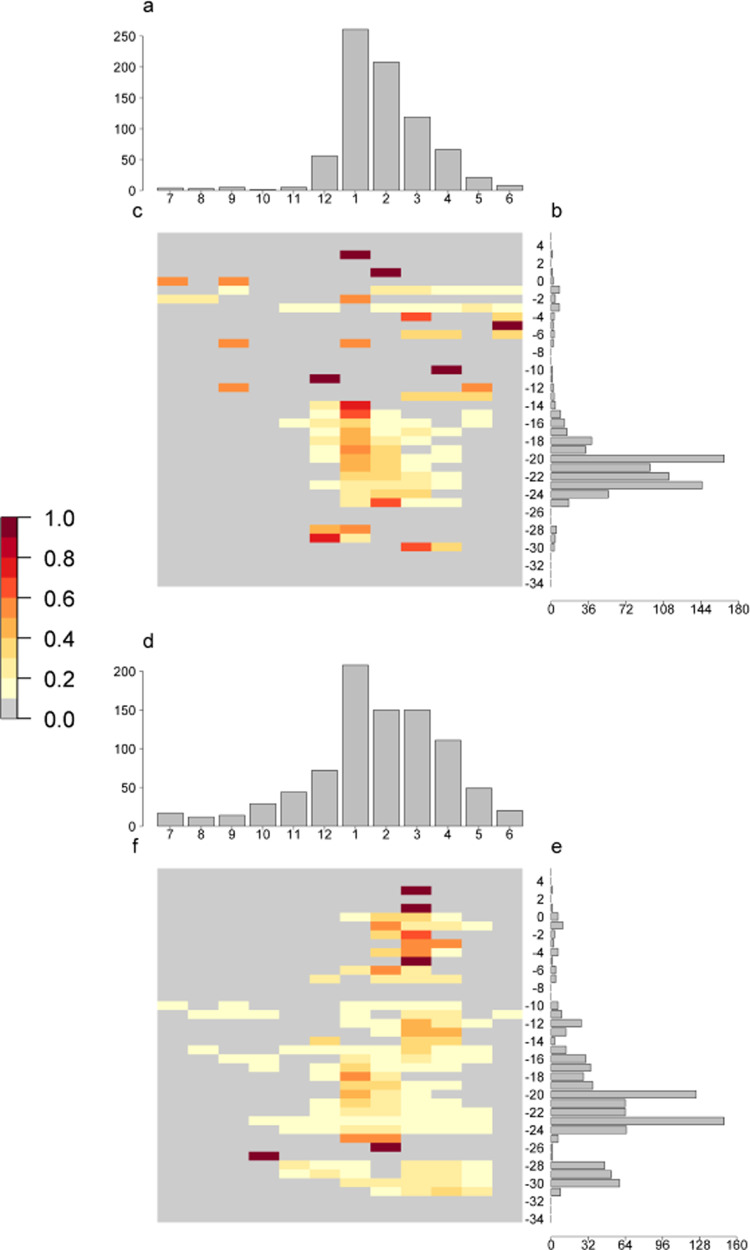
The proportion of human and NHP reports occurring each month by 1° latitude across Brazil. **A** The number of human YF reports by both across Brazil, **B** the proportion of human reports occurring at each latitude, **C** the number of human YF reports by 1° latitude. **D** The number of NHP YF reports by both across Brazil, **E** the proportion of NHP reports occurring at each latitude, **F** the number of NHP YF reports by 1° latitude. Shade of cell refers to the proportion of reports occurring at the latitude.

YF reports were highly seasonal in both humans and NHPs, though specific patterns differed slightly (Fig. 1). Human YF reports are minimal throughout much of the year, June–November, but increase rapidly in December to a peak in January before decreasing towards minimum values in May. In contrast, NHP reporting has a lower seasonality amplitude—with cases reported throughout the year at a background level. Cases increase from October, with a similarly timed peak in January. This remains stable for February and March, before descending to background levels in June.

The vast majority of human reports of YF occur between −18° and −24° latitude, whereas NHP reports have a more widespread distribution, with less clustering and substantial numbers further south at −28° to −30° latitude.

### Model fits and comparison of agricultural seasonality and climate/vegetation

Model fits varied across all covariate inclusions and classification report types. Generally, AUC scores for out-of-sample predictions of reports of human cases and of both human and NHP cases were higher than those of reports of NHP cases alone (Table 1). The best performing model, as measured by the Brier score, was 15 (Table 2). Model OHAC contained all covariate groups. The best-fitting model which did not include agricultural seasonality was OHC, ranked 5th. The out-of-sample AUC for human reports of YF varied from 0.80 (0.73–0.87) in model O, to 0.93 (0.90–0.96) in OHAC (Table 1). AUCs for NHP reports of YF were lower, ranging from 0.78 (0.75–0.82) in model A, to 0.92 (0.90–0.94) in models HAC and OHAC. Municipalities that had both human and NHP reports of YF had out-of-sample AUCs ranging from 0.73 (0.69–0.77) for model A, to 0.84 (0.81–0.87) for the model OHAC.

**Table 1:** Human and NHP YF report models by covariate grouping.

Agriculture output (O)	Host covariates (H)	Agriculture seasonality (A)	Climate/vegetation seasonality (C)	Human report AUC	NHP report AUC	Both report AUC	Brier score	ID	Overall rank
1	0	0	0	0.80 (0.73–0.87)	0.78 (0.75–0.82)	0.73 (0.69–0.77)	0.019291	O	13
0	1	0	0	0.84 (0.78–0.90)	0.81 (0.78–0.84)	0.74 (0.70–0.78)	0.019051	H	11
1	1	0	0	0.86 (0.81–0.91)	0.84 (0.81–0.87)	0.78 (0.74–0.81)	0.019493	OH	15
0	0	1	0	0.76 (0.71–0.81)	0.84 (0.81–0.86)	0.69 (0.66–0.72)	0.019302	A	14
1	0	1	0	0.84 (0.79–0.89)	0.86 (0.83–0.89)	0.79 (0.76–0.82)	0.017762	OA	5
0	1	1	0	0.88 (0.84–0.92)	0.90 (0.89–0.92)	0.79 (0.76–0.82)	0.018576	HA	9
1	1	1	0	0.89 (0.85–0.94)	0.90 (0.89–0.92)	0.83 (0.80–0.86)	0.01708	OHA	2
0	0	0	1	0.85 (0.80–0.90)	0.86 (0.84–0.89)	0.70 (0.66–0.73)	0.019285	C	12
1	0	0	1	0.89 (0.85–0.93)	0.88 (0.86–0.90)	0.78 (0.75–0.81)	0.018159	OC	7
0	1	0	1	0.91 (0.88–0.95)	0.91 (0.89–0.93)	0.79 (0.76–0.82)	0.018399	HC	8
1	1	0	1	0.92 (0.89–0.95)	0.90 (0.88–0.93)	0.81 (0.78–0.84)	0.01784	OHC	6
0	0	1	1	0.87 (0.82–0.91)	0.88 (0.85–0.90)	0.77 (0.75–0.80)	0.018636	AC	10
1	0	1	1	0.90 (0.86–0.94)	0.89 (0.87–0.91)	0.82 (0.79–0.85)	0.01757	OAC	3
0	1	1	1	0.92 (0.89–0.96)	0.92 (0.90–0.94)	0.83 (0.80–0.86)	0.01772	HAC	4
1	1	1	1	0.93 (0.90–0.96)	0.92 (0.90–0.94)	0.84 (0.81–0.87)	0.01708	OHAC	1

The presence, 1, or absence, 0, of covariate groupings is shown with the corresponding out-of-sample AUC, Brier score and overall model rank. The best models according to Brier score are highlighted.

**Table 2:** Absolute total deviances between YF reports and within-sample model predictions (for models fitted to all the data) by covariate grouping.

Covariate groupings	Monthly difference from data (Total YF reports—total model predictions)		
	Human	NHP	Both
OHA	295.4	249.4	91.3
OHC	440.1	414.8	117.3
OHAC	278.7	260.0	71.2

Results are shown for the best fit model including agricultural (but not climate) seasonality (OHA), climate (but not agricultural) seasonality (OHC) and both forms of seasonality (OHAC).

**Fig. 2:**
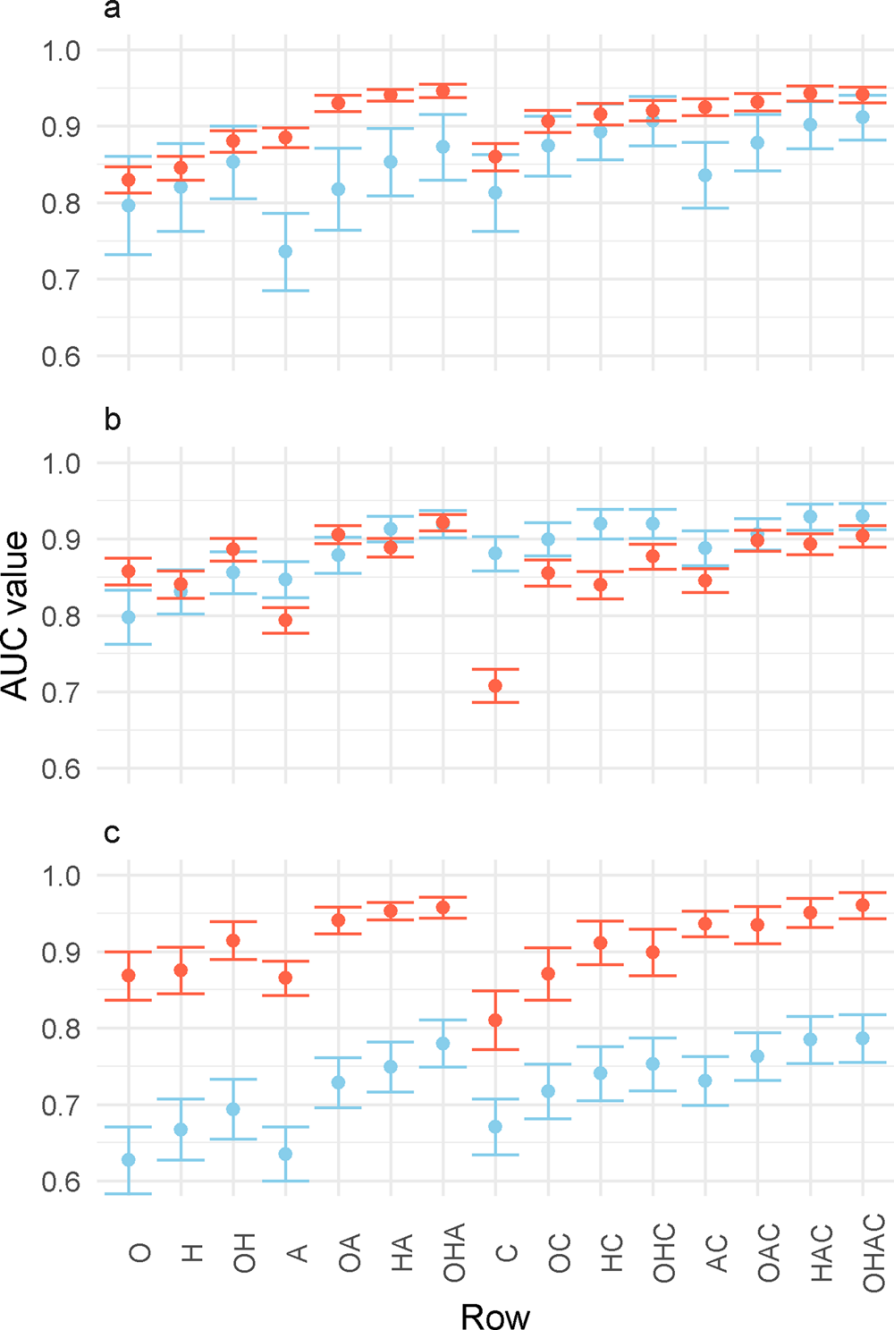
Comparison of training and validating AUC values for human, NHP and municipalities with both. AUC values for the classification of a municipality as having (**A**), human YF report (**B**), NHP YF report and (**C**) human and NHP YF report. The *x*-axis numbers refer to the models found in Table 1. Red refers to the training AUC value and blue the validation AUC value. Error bars represent the 95% confidence intervals of the prediction, calculated from the 100 out-of-sample validation AUC values for each model formulation. *N* = 100 out-of-sample calculations of the AUC for 15 independent models.

Out-of-sample predictive performance, as calculated using a spatial-block bootstrapping method was overlapping or only slightly lower than within-sample performance for predicting the human and NHP reports, but slightly worse for predicting both reports (Fig. 2). Out-of-sample performance tracked within-sample performance for all models.

### Seasonal trends in model predictions

**Fig. 3:**
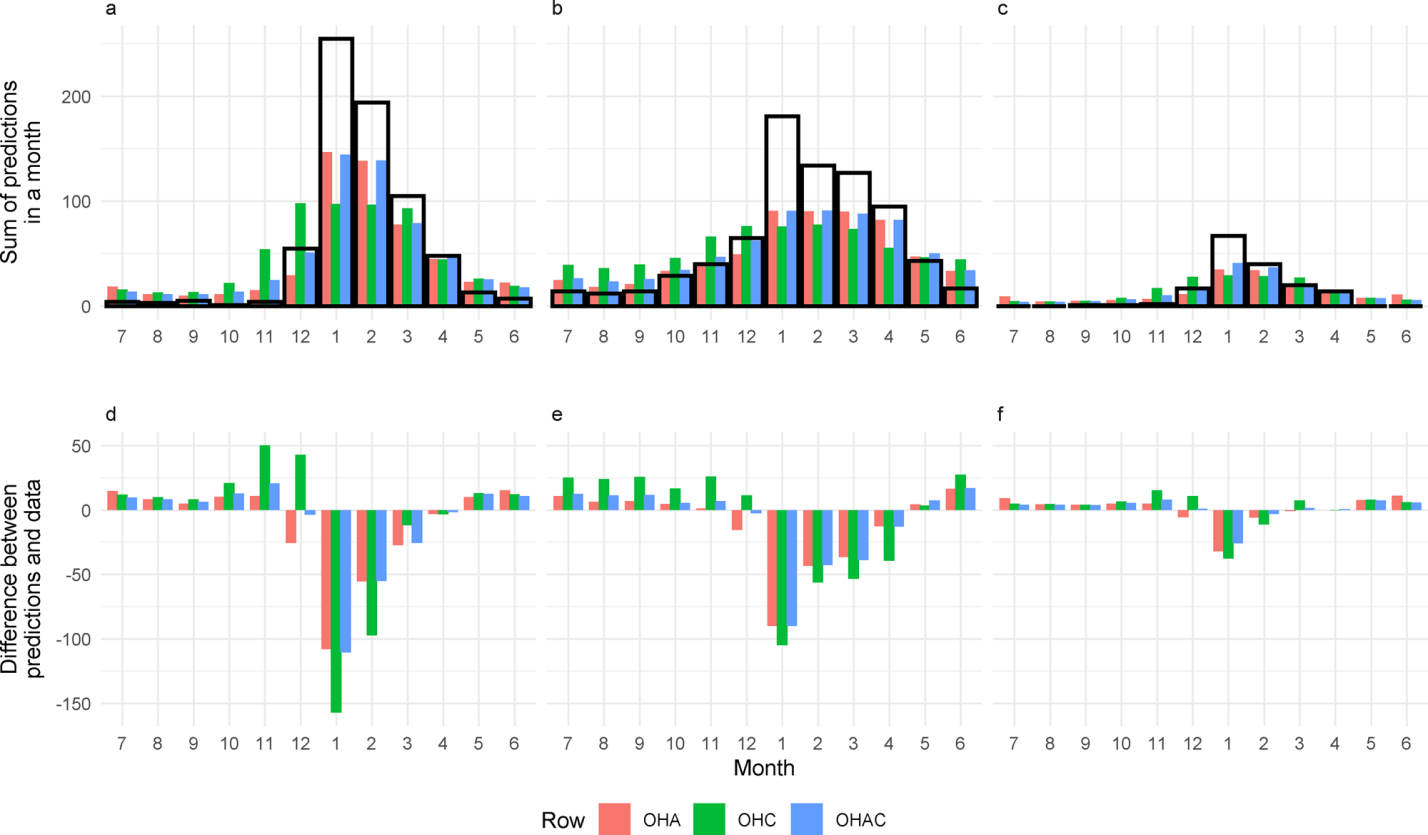
Comparison of monthly model predictions and the data for models including seasonality of climate, agriculture and both. Total monthly YF reports and in-sample model predictions **A, D** for humans, **B, E** NHPs and **C, F** both classifications. The top row (**A, B, C**) depicts the overall monthly data and model predictions for each classification type. The black rectangles indicate the data. The bottom row (**D, E, F**) show the residuals. Results are shown for the best fit model including agricultural (but not climate) seasonality (model OHA), climate (but not agricultural) seasonality (model OHC) and both forms of seasonality (model OHAC). Within-sample predictions are shown. Red refers to the OHA model predictions, green the OHC and blue the OHAC.

While all covariate groupings captured the monthly seasonality of YF to a degree, they did so at differing levels of accuracy. The seasonality of human YF reports was generally better reproduced (correlation of 0.80 in the best fit climate/vegetation seasonality model to 0.99 in the best fit including both types of seasonality) than the seasonality of NHP reports (0.83 in the climate/vegetation seasonality model to 0.95 in the models that included the agriculture of seasonality) or of reports of both human and NHP cases (0.80 in the agricultural seasonality model to 0.97 in the OHAC model) (Table 2, Table 3, Table 4 and Fig. 3).

**Table 3:** Pearson’s correlation values comparing within-sample model predictions (for models fitted to all the data) with the data by covariate grouping.

Covariate groupings	Monthly predictions correlation		
	Human	NHP	Both
OHA	0.99	0.95	0.94
OHC	0.80	0.83	0.80
OHAC	0.99	0.95	0.97

Results are shown for the best fit models including agricultural (but not climate) seasonality (OHA), climate (but not agricultural) seasonality (OHC) and both forms of seasonality (OHAC).

**Table 4:** Covariate groupings for statistical modelling.

Groupings	Covariates	Monthly variation in covariate	Number of covariates
Agricultural output (O)	Number of farms producing each crop type	No	8
Host demographics (H)	Number of NHP species, proportion of total human population working in agriculture, log of rural human population	No	3
Agricultural seasonality (A)	Binary (0, 1) indicator for planting and harvesting of the 8 crop types measured in the agriculture output	Yes	16
Climate/vegetation seasonality (C)	Rainfall, day and night temperature, the temperature range and the EVI, as well as the 1 and 2 month lagged values of these covariates	Yes	15

The best fit models that included the seasonality of agriculture provided a substantially better fit to the seasonality of human reports (Table 2, Table 3 and Fig. 3). In particular they more accurately captured the magnitude of seasonality—something that the best fit model that only included the seasonality of climate/vegetation failed to account for. Models generally underpredicted reports of YF in months of heightened transmission, and marginally overpredicted during the “low season” (Fig. 3). While no models captured the true magnitude of the peak of the epidemic, models that included agriculture seasonality more accurately represented the data than those without.

At a national level, there is significant seasonality in YF reports (human, NHP and both), with 79.8% of all reports occurring January–March, and a minimum of 1 report in October, and 255 in January. The probability of a human report is minimal from July to October for all models and the data, while in November the best fit climate/vegetation seasonality model predicts a substantial increase in reports this is not reflected in the data or the other model predictions. A rise in actual reports, from 4 to 55, and predicted reports in December, with the climate/vegetation seasonality model over-predicting the number of reports. January sees a significant increase in the reporting of cases, rising from 55 to 255 reports, followed by a fall to 194 in February, a trend which is accurately followed in all models’ predictions, apart from the climate/vegetation seasonality model which underpredicts substantially.

NHP reports follow a less strongly seasonal pattern than human reports, with higher levels of reporting seen across the year, with the minimum of 12 reports occurring in August, and the maximum of 181 in January. Model predictions follow a similar pattern to predicting to the human reports, with the climate/vegetation model predictions consistently over-predicting the “low-season” months of June–December, and under-reporting the peak of January–April, with other models generally performing well.

### Geographical distribution of YF reports

**Fig. 4:**
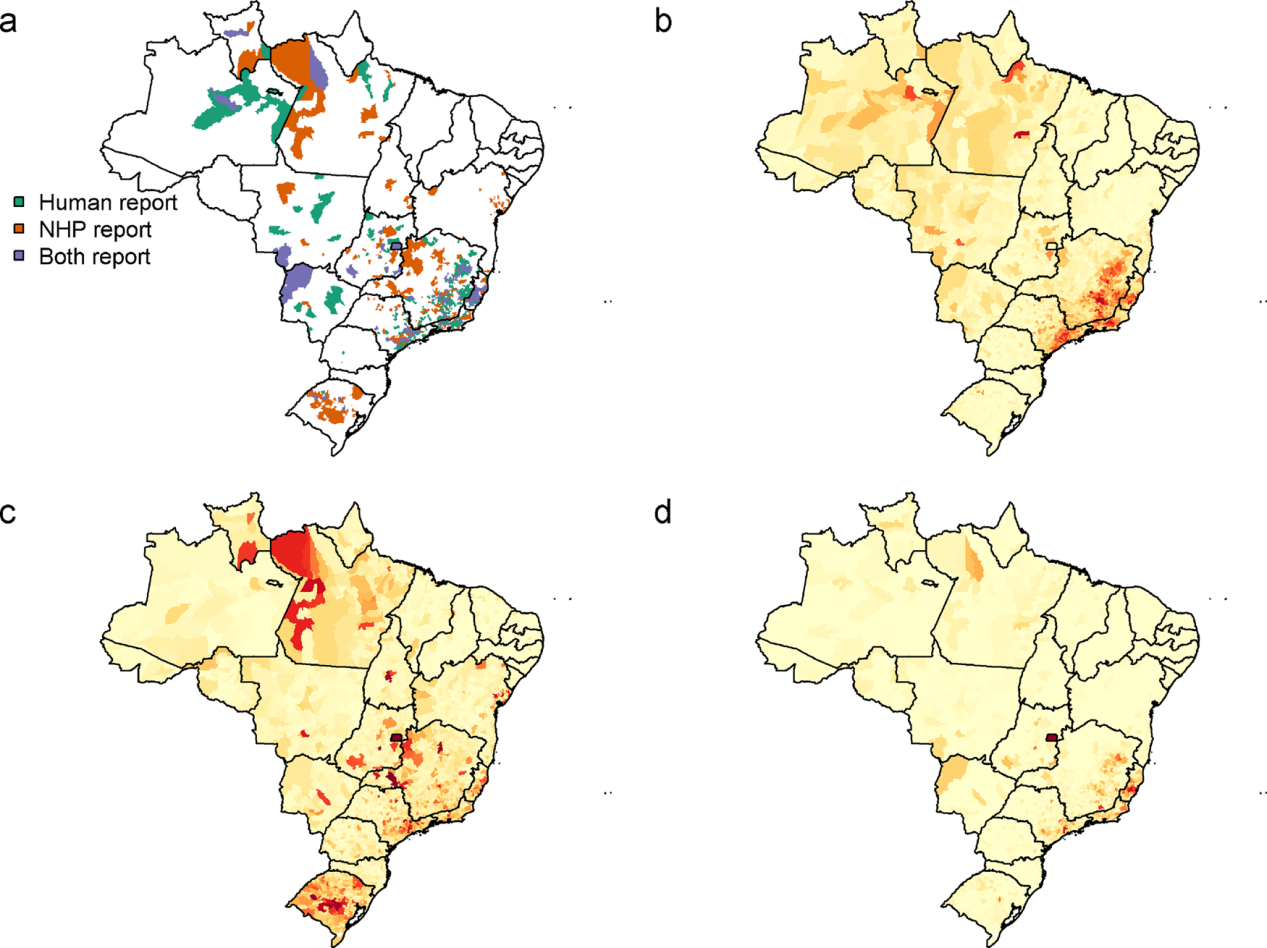
Data and predictions of YF cases in humans, NHPs and both across Brazil. **A** Aggregate reports of the data for human, NHP and both reports model predictions for the probability of classifying an administrative location as **A** only having human reports, **C** only NHP reports and **D** both human and NHP reports. Model predictions are from the best fit model with all covariates (model OHAC). Colours indicate the probability of presence for each of the report type in figures **B–D**.

Reports of YF in all classifications are found throughout much of the country, with the exception of the North East of Brazil (Fig. 4).

Notable hotspots for human reports are seen in the South-Eastern Atlantic states of Brazil, Western and Amazonian states. NHP reports are more widely spread throughout the country, with reports in states without human cases such as Bahia and Tocantins. Municipalities with both human and NHP reports reflected the distributions of human and NHP reports, with much of Espírito Santo and large areas of São Paulo state recording both human and NHP reports.

The best fit model (OHAC) reproduced all these patterns well. The predicted pattern of human reports largely matched the data, with the exception of predictions of higher in the North states that constitute part of the Amazon where cases have not been reported in this time period.

### Variable importance comparisons for best-fitting models

We assessed variable importance ranks for the best-fitting models with only agricultural seasonality (OHA), only climate seasonality (OHC) and with both (OHAC).

**Fig. 5:**
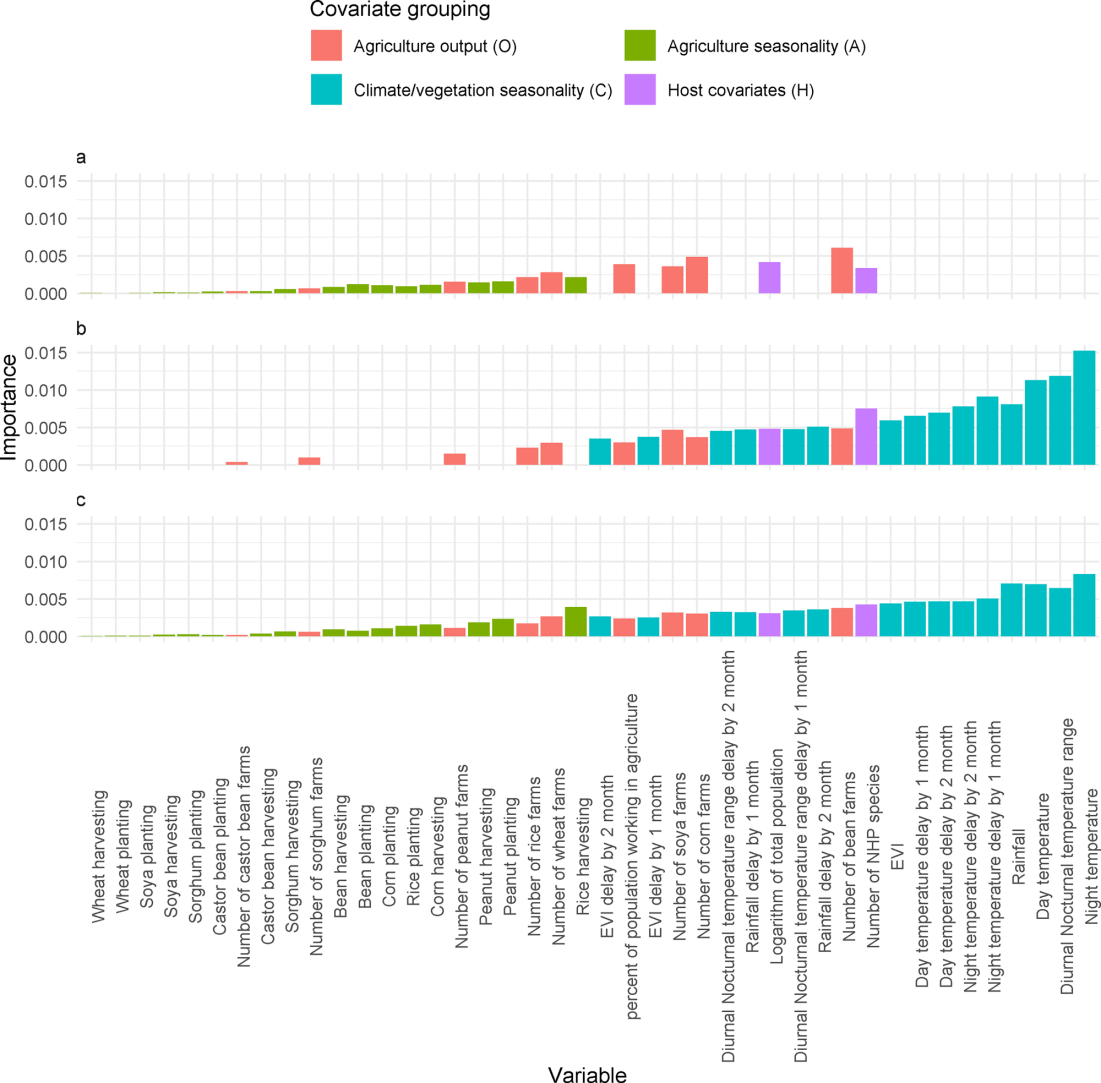
Covariate variable importance for models looking at the seasonality of climate, agriculture and both. Variable importance values for **A** the model with agricultural seasonality but not climate seasonality, model OHA, **B** the model with vegetation/climate seasonality but not agricultural seasonality, OHC and **C** the model with both agriculture and vegetation/climate seasonality, model OHAC. Variables are omitted when note present in the model.

For models which included vegetation/climate grouping, these had high levels of importance attributed, similarly host covariates were ranked favourably in all models. Agriculture output and agriculture seasonality were not found to have high values in permutation importance (Fig. 5).

In the OHAC model, the top performing covariates were related to temperature and rainfall, with Night Temperature particularly important (any its value in the last and 2 months ago similarly ranked highly). The most important agricultural output covariates were the number of bean, corn and soya farms and the agricultural seasonality covariates that had the most influence was rice harvesting and peanut planting.

## Discussion

We have identified the highly seasonal nature of YF reporting in both humans and NHP’s, as well as demonstrating the relative predictive power of utilising covariates related to the seasonality of climate and the seasonality of agriculture. All model fits accurately captured the seasonality of reporting in humans and NHP’s, though models fit to reports of YF that included humans performed significantly better. Models that included the seasonality of agriculture had a significant and substantial improvement in their ability to predict human reports (aggregate monthly correlation: 0.99 vs 0.80) (Table 3 and Table 1). Our findings illustrate the importance of the seasonality of exposure, and that it is not necessarily just an increased viral transmission in zoonotic reservoir hosts which leads to spillover, but also an increased interaction with the sylvatic cycle. In addition to this we have highlighted the individual role of different crop types such as peanut and bean planting/harvesting on increasing the probability of YF reporting.

While the link between agriculture and disease has long been highlighted, there has been little work done on how the seasonality of exposure relates to increased disease transmission, and even less for yellow fever. The increased predictive performance of models fit to the seasonality of agriculture over climate is an important finding for predicting zoonotic spillover into human populations. Despite climate dictating the environmental suitability for vectors, and so likely increasing viral transmission within the sylvatic reservoir host, it is covariates that indicate an increased exposure to the sylvatic cycle that appear more determinant. The importance of human-animal contact in zoonotic spillover has previously been highlighted as a significant determinant of spillover events^22,23^, though this has not been explored in the context of seasonality of agriculture as a driver of exposure for vector-borne diseases.

Although further study is needed to establish a mechanistic understanding of how these agricultural activities increase exposure to the sylvatic cycle, the associations highlighted here represent an important first step. Agricultural activities in Brazil, and much of the world, are rapidly changing. Rising populations and the growing of “cash” crops, such as rice and soya, for exportation are changing the agricultural ecosystem, as well as driving deforestation and general habitat conversion^24^. These changes are likely to lead to both short term effects, and long-term changes in the epidemic and endemic potential of numerous diseases—particularly those with a zoonotic component^18,25,26^. Our findings suggest that these changes, in addition to changing the overall suitability of a habitat, may even change the relative seasonality of spillover. Following the initial disruptions to the sylvatic cycles that are brought on by land conversion, the regular transformation of the landscape, through planting and harvesting, as well as the increased interaction of humans to this habitat appear to increase the risk of YF spillover for several crop types and activities. This “anthropogenic seasonality” may have public health consequences for surveillance and further transmission. Aligned with the strong seasonality typically associated with changes in rainfall and temperature^7,8^, surveillance efforts for YF are generally intensified between December and May in Brazil with priorities shifted towards other activities outside of this time period, a strategy undertaken following a YF case series analysis (1970–2008)^27^. However, if this seasonal spillover is not merely dictated by climate as was previously believed, then human transmission may be occurring undetected at higher levels than currently suspected outside of the traditional seasonal period, especially in endemic zone. Undetected and unopposed spillover into humans additionally raises the risk of establishment in *Aedes aegypti* populations—potentially sparking urban epidemics which have historically spread rapidly, been hard to contain, and reached outside of their country of origin^3,28^.

Despite the substantial model improvements offered when including the seasonality of agriculture, the model OHAC which considered both the seasonality of climate and agriculture, highlighted the variable importance of climate. Without an increased sylvatic transmission of the virus, determined by climate, spillover cannot occur and so still plays an important role in YF transmission in Brazil. Although climate and agriculture are intrinsically linked, with different activities occurring at times where climate favours growing and harvesting, they are not equivalent. In addition to the significant differences in model fit, climate and agricultural activity covariates only show moderate correlation. This is partly because agriculture is not solely decided by the climate, with anthropomorphic adaptations such as the use of irrigation, fertilisers and herb/pesticides allowing for an ever-increasing detachment from seasonal farming. This, in conjunction with the binary nature of our agriculture seasonality data, and analysis into covariate correlations, variable inflation fractions and exploring the impact of different methodologies, suggests that we are investigating two separate processes and not simply overfitting to climate driven data.

Although in and out-sample AUC values of models fit to human reports of YF were not significantly different (or substantially so), AUC values for predicting both reports showed larger differences, indicating potential overfitting. This suggests that these models do not capture the underlying transmission dynamics distinguishing areas as spillover where both human and NHP cases are founds, an unsurprising finding given the differences expected in human and NHP exposure to the sylvatic cycle. Additionally, this result may relate to the surveillance of NHP cases. As the number of humans entering habitats suitable for NHP’s and sylvatic mosquitoes increases, then there will be an increased observation of NHP’s and so the probability of detecting an NHP YF report will increase. Therefore, the relationship we have captured may be partially related to a seasonal increase in surveillance, a finding which could be used to correct for seasonal variations in NHP report detection biases related to exposure to sylvatic habitats.

Human reports of YF, geo-located to the site of infection rather than the site of reporting, are representative of the environmental and climatic covariates associated with transmission. Unfortunately, reporting of NHP YF brings substantial heterogeneities in surveillance sensitivity across Brazil, with some regions not reporting these events officially, despite their occurrence. While it may seem intuitive for the health system to specifically deal with human health, regions where there are partnerships with other governmental organisations are better at detecting these events, and so improve human health by considering the entire ecology of disease transmission, not just the human component. The heterogeneities in NHP reporting may additionally explain why models that included human reports of YF performed better with human reporting less influenced by heterogeneity in sensitivity. Furthermore, in some areas the reporting of epizootic events is directly related to human reports of YF, with zoonotic surveillance intensified following the reporting of a human case of YF. While this follows a sensible protocol for disease surveillance and response, it introduces additional surveillance biases into our model, which may affect the relationships between NHP YF reports and the environment/climate that we are trying to capture.

Future work may be able to expand this analysis through increased detail on the seasonality of agricultural activities and reporting. Here we have only been able to collate presence/absence for limited crop types at the first administrative division, which despite its limitations still offers substantial improvements to models (Table 1 and Fig. 3*)*. Additional quantification of the scale of agricultural activities, additional crop types, and their relationship with sylvatic habitats at a higher spatial resolution may reveal further relationships within these agriculture landscape mosaic and YF reporting. This expansion would rely on further geo-localisation of reports, and so in order to increase the accuracy and applicability, the collection and reporting of coordinates of YF reports is vital for improved data and predictions.

In conclusion, our analysis represents an important first investigate the relationship of the seasonality of agriculture and yellow fever, as well as other arboviruses. By identifying the types of agriculture and crops associated with YF transmission, this work has direct and immediate applicability. Through targeting vaccination and surveillance activities towards areas, and time-periods, most at risk of spillover, we can more accurately and effectively prevent human YF before it occurs. This increased understanding of YF spillover is particularly important in the context of limited resources^29^, and a globally changing epidemiology of YF^30^, and the increased risk of international exportation that these bring^28^.

## Methods

### YF reports

YF case data for humans and NHP’s were provided by the Brazilian Ministry of Health at the municipality level for all cases recorded between 2003 and 2018. Cases were anonymised and included a municipality identification number, municipality name, and the date of symptom onset. There were 2423 human cases of YF in the original dataset; of these 10 did not contain a date, 18 could not be geo-located, leaving 2395 cases. These 2395 cases translated to 694 monthly occurrences of YF across 434 unique municipalities.

Case data for NHP’s contained a municipality identification number, municipality name and the data of epizootic event discovery. There were 3209 NHP epizootic events confirmed by either laboratory or epidemiologic link criteria, of which all could be identified at the municipality level, and with a date—though 10 occurred before 2003, leaving 3199 cases. This led to 771 monthly occurrences in 409 unique municipalities.

Monthly reports of YF were aggregated over the time period 2003–2018 due to the relative scarcity of YF reports on an individual annual basis. Thus, the final dataset consisted of occurrence (coded as a binary 0/1 variable) of YF for each of the 12 calendar months and each municipality. Municipality represents the second administrative level in Brazil.

This approach was taken rather than using the number of cases due to the large uncertainties in reporting and detection of YF cases. Due to the presence of asymptomatic infection, and non-specific symptoms in mild cases, in addition to the rural locations and issues related to diagnosis, case numbers represent just the tip of the iceberg, and potentially do not indicate the magnitude of actual transmission, particularly in endemic settings {Monath, 2006 #516}. Furthermore, the differences between regions in their surveillance are likely to be inconsistent. However, by modelling the presence/absence of a YF report in a month, this approach is more robust to these issues, as it only takes a single report of YF during the time period to be classed as a province with YF presence.

### Host demographics

NHP species distribution maps were obtained from NatureServe^31^. This data were available as demarcations of distribution, which was geo-located to the municipality level and used to calculate the number of NHP species present in each location.

Data on the population of each municipality and the proportion of the population working in agriculture were obtained from Instituto Brasileiro de Geografia e Estatística (IBGE). Available from: https://www.ibge.gov.br/estatisticas-novoportal/downloads-estatisticas.html.

### Seasonally varying agricultural activity

Information on agricultural activities (planting, harvesting and planting or harvesting) at the state (first administrative level) was extracted from an agricultural calendar published by Companhia Nacional de Abastecimento (Conab) in conjunction with the Ministério da Agricultura, Pecuária e Abastecimento (Mapa) in Brazil^32^. This provided data on a monthly basis for 15 crops in Brazil. This information was tabulated as a dataset of monthly presence and absence (0/1) of planting and harvesting for each crop.

Of these, eight were chosen for further analysis due to the number of farms producing the crop type: peanuts, rice, the common bean, castor beans, corn, soya, sorghum and wheat. These eight crop types represent 16 binary covariates of planting and harvesting.

### Agricultural output

Information on agricultural output of Brazil at the municipality level is provided by the “2017 Agricultural, Forestry and Aquaculture Census” in a variety of formats at their portal https://censoagro2017.ibge.gov.br/templates/censo_agro/resultadosagro/agricultura.html. This provided the number of farms producing each of the eight crop types that seasonal agriculture data were available for.

### Seasonally varying climate and vegetation

Data on temperature^33^, vegetation (as measured by the Enhanced Vegetation Index (EVI)^34^), and rainfall^35^ were spatially aggregated from their original resolution, of between 1/120 and 1/12 degree, by calculating population-weighted means, based on the population distribution from LandScan 2015^36^ for each municipality in Brazil. Multi-year averages (over 2003–2016) were calculated for each calendar month of the year and municipality.

### Multi-collinearity

RF algorithms automatically reduce correlation amongst the trees by performing a search over a subset of variables as opposed to searching across all variables, with previous research showing that with tuning of the number of trees and depth, RFs are capable of dealing with datasets that have a high degree of correlation^37–39^.

**Fig. 6:**
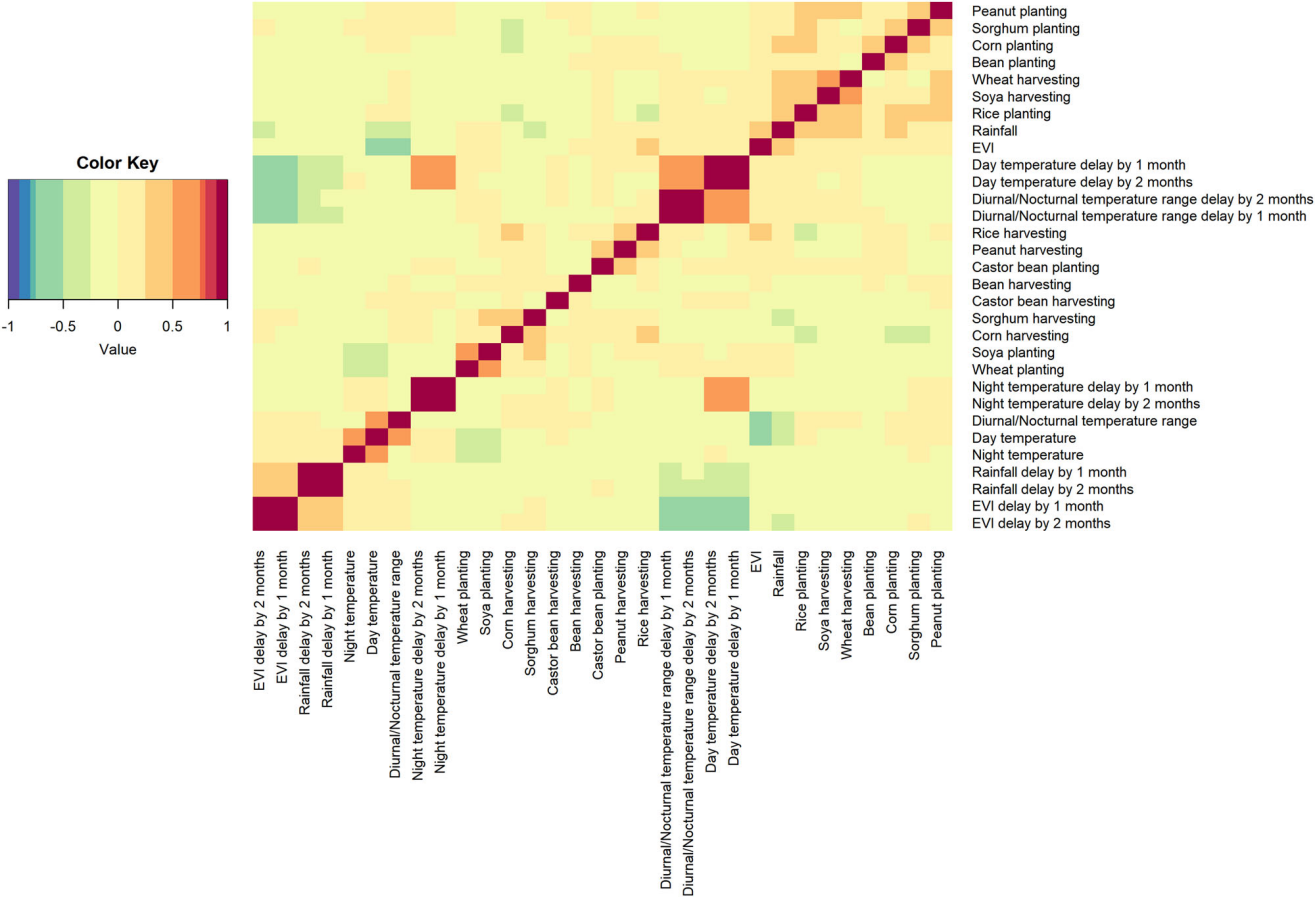
Heatmap of the correlation (−1 to 1) of agriculture seasonality and climate covariates included in the analysis.

Furthermore, we found that none of our seasonally varying agricultural covariates were highly correlated with those in climate/vegetation, lending support to the theory that these are measuring somewhat independent processes and not purely overfitting to patterns in climate/vegetation (Fig. 6).

### Covariate groupings

Agricultural output covariates were included alongside their relevant seasonal variations in agricultural activity, along with covariates related to climate and vegetation, and the number of NHP species, the proportion of the population working in agriculture and the log of human population. In order to increase the relevance of model findings, the dataset was ordered to follow the Brazilian YF surveillance period of July–June.

These were grouped into four classes and all possible combinations of these four classes were investigated, for a total of 15 models (see the SI for a full list of covariates). For the purpose of comparison, covariates were standardised to the have zero mean and unit standard deviation before being used in the random forest models.

### Random forest models

Random Forests (RF) are a machine learning ensemble method which use covariates to explain patterns in data but work by creating a series of decision trees to explain the results^40^. These “trees” are then aggregated, and the mean taken produce a “forest”. These can provide substantial improvements in accuracy over traditional regressions, in addition to accounting for both interactions and non-linear relationships^41^. Random forest modelling was carried out using the Ranger package^42^ in the statistical programming language R^43^, version 3.5.1.

We used the RF models to classify municipalities into one of four categories, no reports of YF, human reports of YF, NHP reports of YF or both, for each month. Permutation variable importance^44^ and partial dependency plots^45^ were calculated for each model to assess the contribution of individual covariates to predicted YF risk.

Model fit for each classification type was assessed by the out-of-sample area under the receiver operating characteristic curve (AUC), a measure of sensitivity and specificity, and the overall model performance rank by the out-of-sample Brier score^46^. The Brier score is a way of modelling the accuracy of probabilistic predictions when outcomes are mutually exclusive, with the lowest score indicating the best set of predictions^47^.

### Out-of-sample validation

Out-of-sample predictive ability was assessed using a spatially disaggregated form of cross-validation called spatial-block bootstrapping. A 5° × 5° grid of longitude and latitude was constructed, and municipalities assigned to grid squares using their centroid coordinates. Grid squares were randomly sampled from this grid with replacement to produce a training dataset of the same size as the original but comprising of 60–70% of the municipalities. The remaining 30–40% of municipalities were used as a validation set. This was repeated 200 times to produce 200 different training and validation datasets

Models were fitted to the training dataset and used to predict the validation dataset, with predictions being assessed via the out-of-sample AUC. This was repeated 200 times with different block bootstrapped training and validation sets. The average AUC across all 200 samples was then taken to ascertain the out-of-sample predictive performance of the models. See Supplementary Material for further details.

### Reporting summary

Further information on research design is available in the Nature Research Reporting Summary linked to this article.

## Supplementary information

Supplementary Information

Reporting Summary

## Data Availability

The underlying data used are anonymised and provided with the code in a repository found at https://github.com/arranhamlet/YF_agriculture_seasonality^48^.

## Code availability

Example code how to load in the data, run the models and produce some of the figures and tables is available at https://github.com/arranhamlet/YF_agriculture_seasonality^48^.
