# Supplementary material for: Seasonality of agricultural exposure as an important predictor of seasonal yellow fever spillover in Brazil
Source: Nat Commun. 2021 Jun 15;12:3647. doi: 10.1038/s41467-021-23926-y (PMC8206143; doi:10.1038/s41467-021-23926-y)
Supplement: Supplementary file 1 — Supplementary Information [file 41467_2021_23926_MOESM1_ESM.pdf]

Supplementary Note 1: Variable importance and partial dependence plots

Variable importance is a calculation of the positive effect of predictive performance [1], and allows us to “rank” variables by their contribution to the model. This can highlight variables that are of particular importance to predictive accuracy. The variable importance for all variables included in the full model, which comprises of every covariate in the dataset, is shown for the model fit to human reports and non-human primate (NHP) reports.

As RF models allow for non-linear relationships, we can also calculate how individual variables influence the outcome, a report of YF, over a range of values. This has been calculated for each variable in the full model fit to human reports and NHP reports. Variables related to the seasonality of agricultural activities (planting and harvesting) are plotted only at two points, 0 (activity not occurring) or 1 (activity occurring).

All covariates were scaled through the following formula,

$$z = \frac{x - \mu}{\alpha},$$

where z, is the standardised value, x, the pre-standardised value, μ, the mean of the pre-standardised values and α, the standard deviation of the values, to allow for direct comparison of variable importance and partial dependence plot.

Variable importance for the best fit models

Here we have described the partial dependence plots (PDP) of the top 50% of variable importance for the best performing model that included only seasonality of agriculture (model 7), seasonality of vegetation/climate (model 11) and the model which included both (model 15). Partial dependence plots show how these covariates influence the outcome across their range of values (Supplementary Figure *1*)

For model 15, generally, following an initial dip for the number of bean and corn farms, increases in the number of farms are associated with increased probabilities of classifying a municipality has possessing a YF reports in NHP’s and both human and NHP, with human reports either remaining relatively unaffected or decreasing over the range of values. The number of NHP species is associated with large increases in the probability of classifying a municipality as reporting all report types of YF, apart from an initial dip in the probability of an area being classed as reporting both human and NHP cases, but quickly plateaus around 8 species. An initial sharp reduction in the association with YF reports is seen for percentages

of the population working in agriculture followed by a slow and minimal increase, while the logarithm of the rural population initially is associated with a decrease in YF reports, it peaks and reduces as values increase, with most of this change due to areas classified as both human and NHP YF report. The harvesting of rice and planting of peanuts is positively associated with all types of YF reporting. As rainfall increases, the probability of YF reports increases, most substantially in areas that report both human and NHP reports. Day temperature's effects on the probability of a report of YF is highest between 20 and 23°C and 37 to 45°C. Increasing night temperatures reduce the probability of a human NHP report, till around 24 °C where it quickly rises. NHP and both human and NHP reports steadily rise over the range of values. Increases in the range of diurnal/nocturnal temperature are negatively associated with all reports until around 15 when it rises. Lower levels of EVI are more positively associated with YF reports, with a slight raise at higher values. Generally, delayed rainfall, temperatures, temperature ranges and EVI follow a similar pattern, with slight deviations between the relative influences on the report classifications, with heightened levels at low values and high values across the ranges.

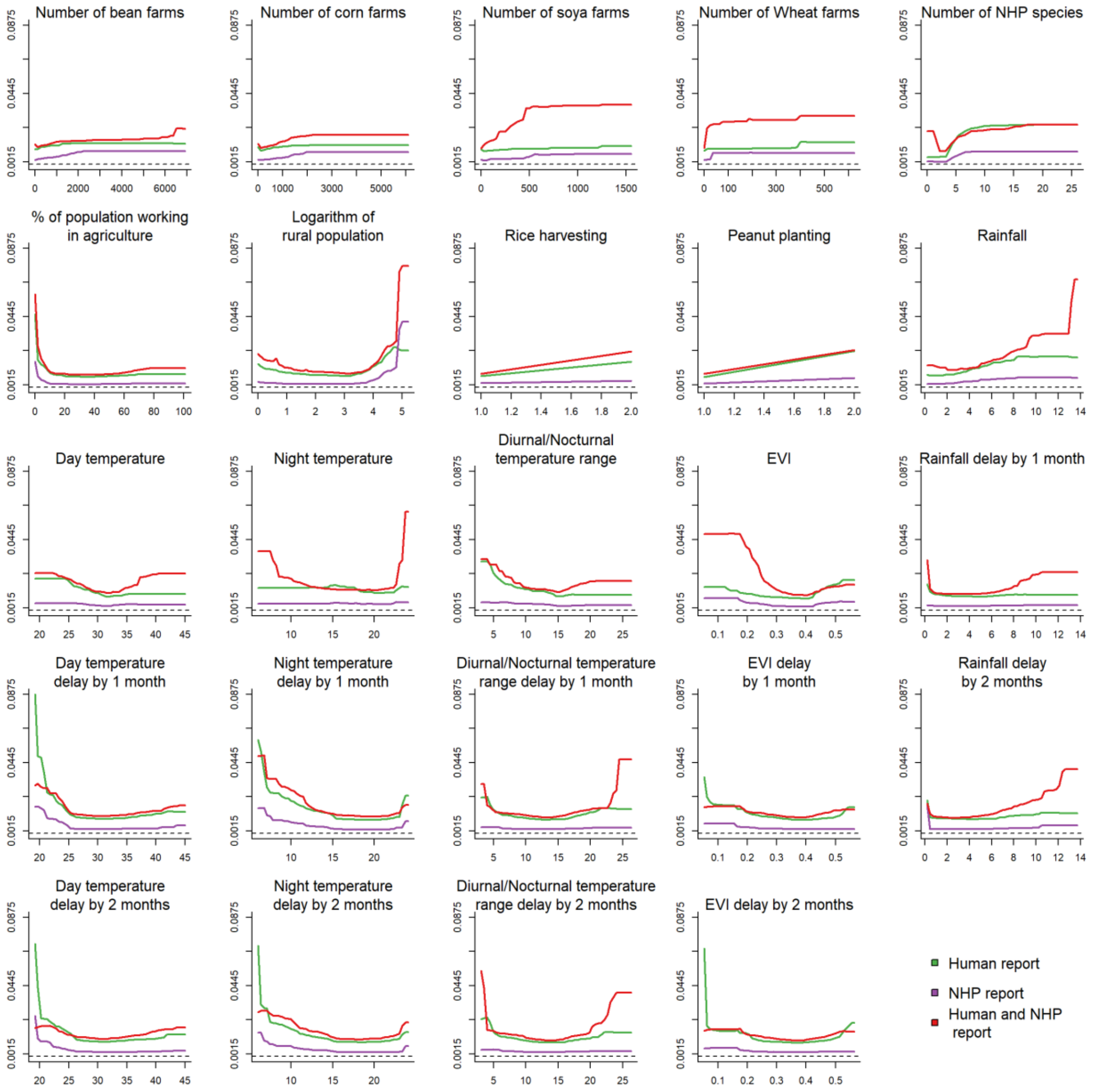

Supplementary Figure 1. Partial dependence plots for the covariates in the top 50% of variable importance of the model that included all covariate groupings. The y axis on the left shows the probability of No report, and the axis on the right the probability of human, NHP and Human and NHP reports.

Supplementary Note 2: Agriculture output covariates

Three measures of agricultural output were available from the "2017 Agricultural, Forestry and Aquaculture Census" [2], the number of farms (Supplementary Figure 2A), the area in hectares (Supplementary Figure 2B) and the quantity in tonnes (Supplementary Figure 2C) produced by each district.

While these measures change the rank in which crop’s are ordered by “highest output”, they are highly correlated (Supplementary Figure *2*D, Supplementary Table *1*).

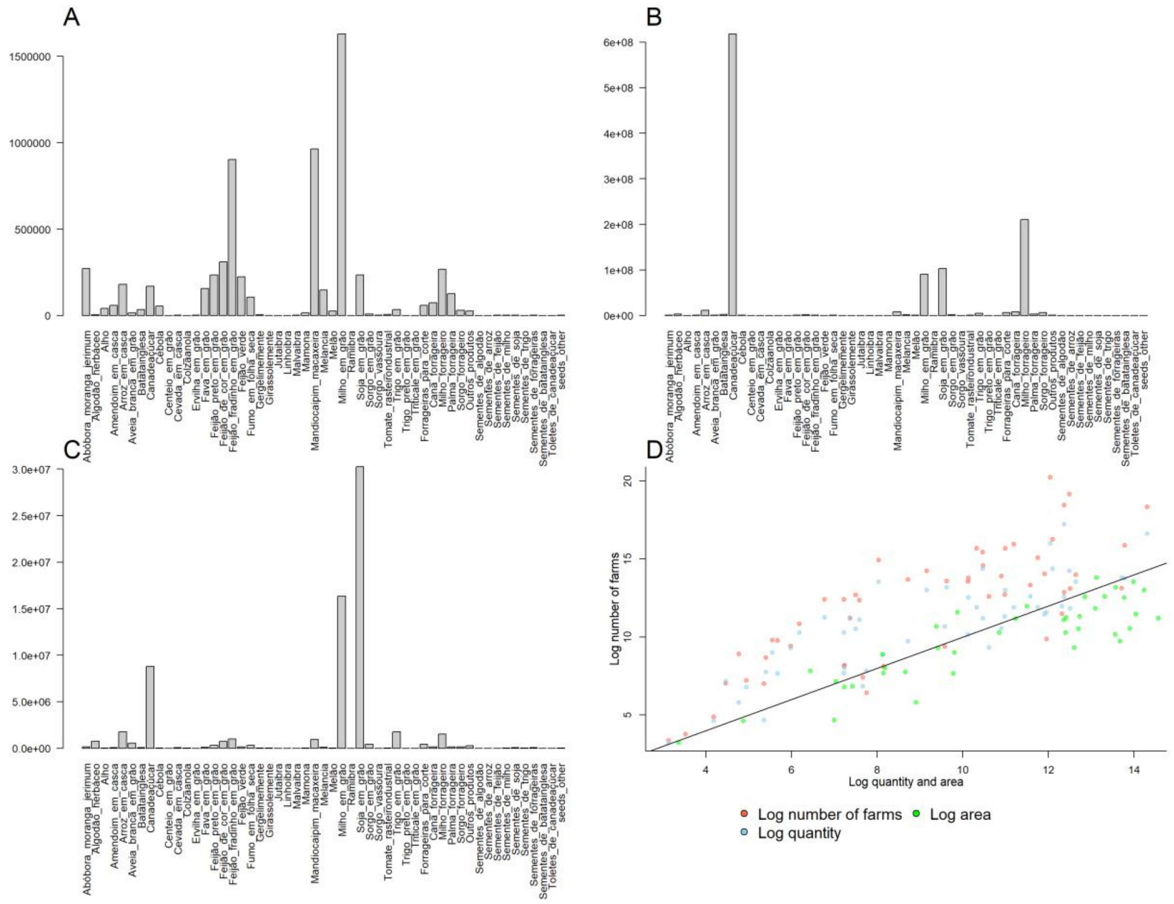

Supplementary Figure 2. Total agricultural outputs for Brazil per crop type for the number of farms (A), the quantity of crop produces in tonnes (B), the area in hectares occupied by cropland (C) and the log of these values plotted against each other (D).

Supplementary Table 1. Correlation between the different measures of Brazil’s agricultural output

Agricultural output types		Pearson’s correlation
Number of farms	Quantity	0.81
Number of farms	Area	0.85
Quantity	Area	0.93

Supplementary Methods 1: Out-of-sample validation through spatial block bootstrapping

Out-of-sample validation: Spatial block bootstrapping

To assess the out-of-sample predictive ability of our models we carried out a form of out-of-sample validation called spatial block bootstrapping.

This was done by overlaying a grid of 5° x 5° longitude of latitude over the study area and assigning provinces to a point based on their centroid coordinates (Figure 1). Following this, random sampling with replacement was used to build a training set of 60-70% of the points that contain an assigned province, the remaining unselected points were assigned to the validation dataset. This was repeated 200 times to generate 200 training sets and 200 validation sets.

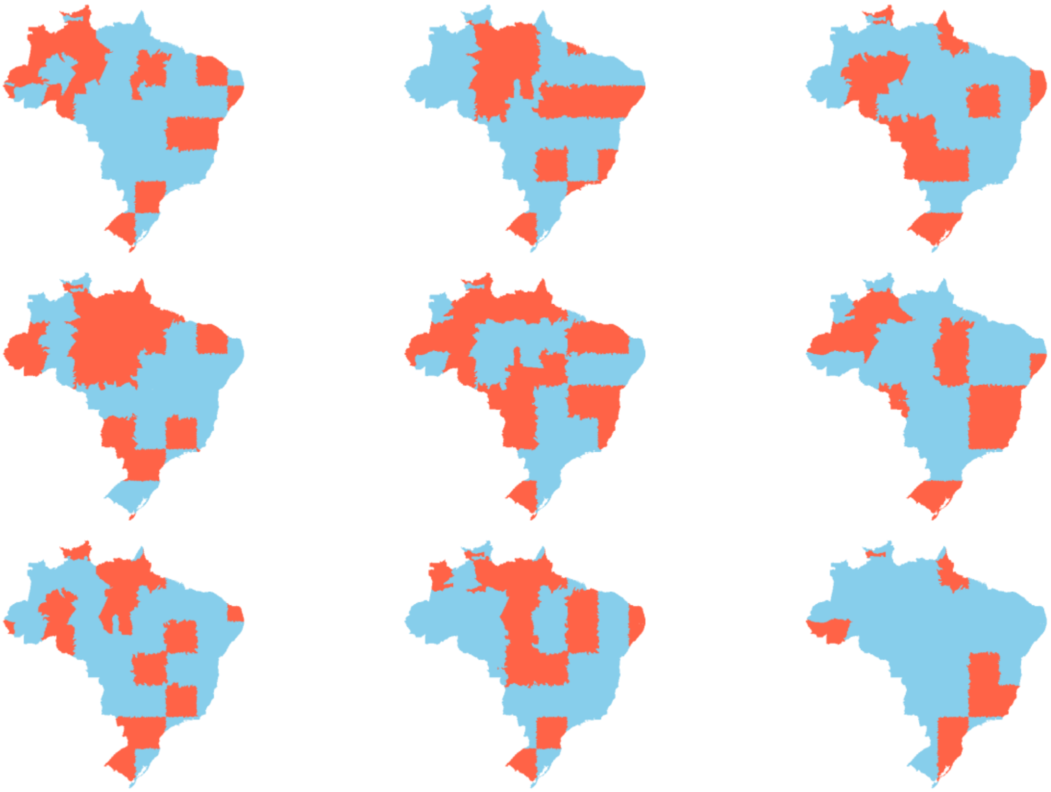

Supplementary Figure 3. Examples of the training (blue) and validation (red) datasets as chosen by random sampling of districts on a grid of 5° x 5° longitude and latitude.

Models were then trained on the training set and predicted to the validation set. These predictions were assessed for model fit using the AUC. Out-of-sample performance was ascertained by the mean model performance across all 200 runs.

Supplementary Tables

Supplementary Table 2. Table of covariates, monthly variation and the source

GROUPING	COVARIATE	MONTHLY VARIATION	SOURCE
**AGRICULTURAL OUTPUT**	Number of peanut farms	No	[2]
	Number of rice farms	"	"
	Number of oat farms	"	"
	Number of bean farms	"	"
	Number of castor bean farms	"	"
	Number of corn farms	"	"
	Number of soya farms	"	"
	Number of sorghum farms	"	"
	Number of Wheat farms	"	"
	% of population working in agriculture	"	Brazilian Ministry of Health
**HOST DEMOGRAPHICS**	Number of NHP species	"	[3]
	Logarithm of total population	"	Brazilian Ministry of Health
**SEASONALITY OF AGRICULTURE**	Wheat planting	Yes	[4]
	Wheat harvesting	"	"
	Bean planting	"	"
	Bean harvesting	"	"
	Corn planting	"	"
	Corn harvesting	"	"
	Rice planting	"	"
	Rice harvesting	"	"
	Peanut planting	"	"
	Peanut harvesting	"	"
	Castor bean planting	"	"
	Castor bean harvesting	"	"
	Sorghum planting	"	"
	Sorghum harvesting	"	"
	Soya planting	"	"
	Soya harvesting	"	"
**SEASONALITY OF VEGETATION/CLIMATE**	Rainfall	"	[5]
	Day temperature	"	[6]
	Night temperature	"	[6]
	Diurnal/Nocturnal temperature range	"	Calculated
	EVI	"	[7]
	Rainfall delay by 1 month	"	
	Day temperature delay by 1 month	"	
	Night temperature delay by 1 month	"	
	Diurnal/Nocturnal temperature range delay by 1 month	"	Calculated
	EVI delay by 1 month	"	

	Rainfall delay by 2 months	“	
	Day temperature delay by 2 months	“	
	Night temperature delay by 2 months	“	
	Diurnal/Nocturnal temperature range delay by 2 months	“	Calculated
	EVI delay by 2 months	“	

Supplementary references

1 Wright, M. N., Ziegler, A. & Konig, I. R. Do little interactions get lost in dark random forests? *Bmc Bioinformatics* **17**, doi:ARTN 145

10.1186/s12859-016-0995-8 (2016).

2 Instituto Brasileiro de Geografia e Estatística (IBGE). *Censo Agro*, 2017).

3 Patterson, B. D. *et al.* (ed NatureServe) (Arlington, Virgina, USA, 2007).

4 Companhia Nacional de Abastecimento (CONAB). Calendário de Plantio e Colheita de Grãos no Brasil 2017. (Agricultural Observatory, 2017).

5 Joyce, R., Janowiak, J., Arkin, P. & Xie, P. CMORPH: A method that produces global precipitation estimates from passive microwave and infrared data at high spatial and temporal resolution. *J. Hydromet* **5**, 487-503 (2004).

6 Garske, T., Ferguson, N. M. & Ghani, A. C. Estimating air temperature and its influence on malaria transmission across Africa. *PloS one* **8**, e56487, doi:10.1371/journal.pone.0056487 (2013).

7 NASA. *Land Processes Distributed Active Archieve Centre (LP DAAC) Vegetation Indices 16-Day L3 Global 1 km (13 A2)*, <http://lpdaac.usgs.gov/get_data> (
