# Supplementary material for: Seasonality of agricultural exposure as an important predictor of seasonal yellow fever spillover in Brazil
Source: Nat Commun. 2021 Jun 15;12:3647. doi: 10.1038/s41467-021-23926-y (PMC8206143; doi:10.1038/s41467-021-23926-y)
Supplement: Supplementary file 2 — Reporting Summary [file 41467_2021_23926_MOESM2_ESM.pdf]

nature research

Corresponding author(s): Arran Hamlet

Last updated by author(s): May 4, 2021

Reporting Summary

Nature Research wishes to improve the reproducibility of the work that we publish. This form provides structure for consistency and transparency in reporting. For further information on Nature Research policies, see our Editorial Policies and the Editorial Policy Checklist.

Statistics

For all statistical analyses, confirm that the following items are present in the figure legend, table legend, main text, or Methods section.

n/a	Confirmed	
☐	☒	The exact sample size (*n*) for each experimental group/condition, given as a discrete number and unit of measurement
☐	☒	A statement on whether measurements were taken from distinct samples or whether the same sample was measured repeatedly
☒	☐	The statistical test(s) used AND whether they are one- or two-sided<br>*Only common tests should be described solely by name; describe more complex techniques in the Methods section.*
☐	☒	A description of all covariates tested
☐	☒	A description of any assumptions or corrections, such as tests of normality and adjustment for multiple comparisons
☒	☐	A full description of the statistical parameters including central tendency (e.g. means) or other basic estimates (e.g. regression coefficient) AND variation (e.g. standard deviation) or associated estimates of uncertainty (e.g. confidence intervals)
☒	☐	For null hypothesis testing, the test statistic (e.g. *F*, *t*, *r*) with confidence intervals, effect sizes, degrees of freedom and *P* value noted<br>*Give P values as exact values whenever suitable.*
☒	☐	For Bayesian analysis, information on the choice of priors and Markov chain Monte Carlo settings
☒	☐	For hierarchical and complex designs, identification of the appropriate level for tests and full reporting of outcomes
☒	☐	Estimates of effect sizes (e.g. Cohen's *d*, Pearson's *r*), indicating how they were calculated

Our web collection on statistics for biologists contains articles on many of the points above.

Software and code

Policy information about availability of computer code

Data collection	Reports of YF in humans and primates was provided by the Brazilian Ministry of Health through personal communication, and all additional data was taken from various online open-source platforms
Data analysis	All data curation and analysis was carried out in the software R version 3.6. The ranger package (version 0.12.1) was used to conduct the random forest models in the main manuscript, and the package gbm (version 2.1.8) for the boosted regression trees. The data and code used, as well as references to all packages, is found at https://github.com/arranhamlet/YF_agriculture_seasonality

For manuscripts utilizing custom algorithms or software that are central to the research but not yet described in published literature, software must be made available to editors and reviewers. We strongly encourage code deposition in a community repository (e.g. GitHub). See the Nature Research guidelines for submitting code & software for further information.

Data

Policy information about availability of data

All manuscripts must include a data availability statement. This statement should provide the following information, where applicable:
- Accession codes, unique identifiers, or web links for publicly available datasets
- A list of figures that have associated raw data
- A description of any restrictions on data availability

The data used to produce these findings can be found at https://github.com/arranhamlet/YF_agriculture_seasonality
NHP species distribution maps were obtained from NatureServe (https://www.natureserve.org/)
Data on the population of each municipality and the proportion of the population working in agriculture were obtained from Instituto Brasileiro de Geografia e Estatística (IBGE). Available from: https://www.ibge.gov.br/estatisticas-novoportal/downloads-estatisticas.html.
Information on agricultural activities (planting, harvesting and planting or harvesting) at the state (first administrative level) was extracted from an agricultural

calendar published at https://www.conab.gov.br/institucional/publicacoes/outras-publicacoes/item/7694-calendario-agricola-plantio-e-colheita
Information on agricultural output of Brazil at the municipality level is provided by the "2017 Agricultural, Forestry and Aquaculture Census" in a variety of formats at their portal https://censoagro2017.ibge.gov.br/templates/censo_agro/resultadosagro/agricultura.html.
Data on temperature (Garske et al., 2013), vegetation (as measured by the Enhanced Vegetation Index (EVI) (NASA)), and rainfall (Joyce et al., 2004) were spatially aggregated from their original resolution, of between 1/120 and 1/12 degree, by calculating population-weighted means, based on the population distribution from LandScan 2015 (Bright et al., 2016)

Field-specific reporting

Please select the one below that is the best fit for your research. If you are not sure, read the appropriate sections before making your selection.

☒ Life sciences ☐ Behavioural & social sciences ☐ Ecological, evolutionary & environmental sciences

For a reference copy of the document with all sections, see nature.com/documents/nr-reporting-summary-flat.pdf

Life sciences study design

All studies must disclose on these points even when the disclosure is negative.

Sample size	No sample size calculations were performed as these are not relevant for random forest or boosted regression tree methods. The 694 occurrences of yellow fever in humans and 771 in non-human primates represents the entire dataset 2003-2018.
Data exclusions	There were 2423 human cases of YF in the original dataset; of these 10 did not contain a date, 18 could not be geo-located, leaving 2395 cases.<br>There were 3209 NHP epizootic events confirmed by either laboratory or epidemiologic link criteria, of which all could be identified at the municipality level, and with a date – though 10 occurred before 2003, leaving 3199 cases.
Replication	The study was repeated using an additional method, boosted regression trees, and findings replicated in out-of-sample predictions using spatial-block bootstrapping
Randomization	Out-of-sample predictive ability was assessed using a spatially disaggregated form of cross-validation called spatial-block bootstrapping. A 5° x5° grid of longitude and latitude was constructed, and municipalities assigned to grid squares using their centroid coordinates. Grid squares were randomly sampled from this grid with replacement to produce a training dataset of the same size as the original but comprising of 60-70% of the municipalities. The remaining 30-40% of municipalities were used as a validation set. This was repeated 200 times to produce 200 different training and validation datasets
Blinding	Blinding is not relevant to this study as it is a statistical modeling study, not involving any data collection or intervention

Reporting for specific materials, systems and methods

We require information from authors about some types of materials, experimental systems and methods used in many studies. Here, indicate whether each material, system or method listed is relevant to your study. If you are not sure if a list item applies to your research, read the appropriate section before selecting a response.

Materials & experimental systems

n/a	Involved in the study	
☒	☐	Antibodies
☒	☐	Eukaryotic cell lines
☒	☐	Palaeontology and archaeology
☒	☐	Animals and other organisms
☒	☐	Human research participants
☒	☐	Clinical data
☒	☐	Dual use research of concern

Methods

n/a	Involved in the study	
☒	☐	ChIP-seq
☒	☐	Flow cytometry
☒	☐	MRI-based neuroimaging
